# Letermovir safety and efficacy for cytomegalovirus prophylaxis in adult Japanese kidney transplant recipients: a multicenter, open-label, noncomparative Phase 3 study

**DOI:** 10.1007/s10157-024-02471-0

**Published:** 2024-04-13

**Authors:** Hideki Ishida, Norihiko Goto, Ryoichi Imamura, Hajime Sasaki, Kohei Unagami, Kenta Futamura, Yoshihiko Murata, Nobuyuki Oshima, Toshiko Eto, Barbara Haber

**Affiliations:** 1 Department of Organ Transplant Medicine, Tokyo Women’s Medical University, Tokyo, Japan; 2 Department of Transplant Surgery and Transplant Nephrology, Japanese Red Cross Aichi Medical Center Nagoya Daini Hospital, Nagoya, Japan; 3 Department of Urology, Osaka University Graduate School of Medicine, Osaka, Japan; 4 Department of Kidney Transplant Surgery, Sapporo City General Hospital, Sapporo, Japan; 5 Merck & Co., Inc., Rahway, NJ USA; 6 MSD K.K., Tokyo, Japan

**Keywords:** Letermovir, Cytomegalovirus, Japanese, Kidney transplant

## Abstract

**Background:**

Letermovir is approved for cytomegalovirus (CMV) prophylaxis in adult allogeneic hematopoietic cell transplantation recipients worldwide and is also approved in the United States for CMV prophylaxis in adult high-risk (D+/R−) kidney transplant recipients (KTRs). The safety and efficacy of letermovir for CMV prophylaxis in adult Japanese KTRs are reported here.

**Methods:**

In this Phase 3, single-arm, open-label study, adult Japanese KTRs with CMV serostatuses D+/R−, D+/R+, and D−/R+ received letermovir 480 mg daily orally within 7 days post-transplant through Week 28. Participants were followed through Week 52. The primary objective was to evaluate letermovir safety and tolerability. Efficacy was a secondary objective, measured by CMV disease, CMV disease or infection requiring intervention, and quantifiable CMV DNAemia. All CMV disease cases were confirmed by an independent adjudication committee.

**Results:**

Among 22 participants (12 were D+/R−) who received letermovir prophylaxis, 20 (90.9%) experienced ≥ 1 AE through Week 28. Most AEs were mild to moderate in severity; no deaths were reported. During the prophylaxis period through Week 28, one transient case of quantifiable CMV DNAemia was detected, but no CMV disease or infection requiring intervention was reported. Through Week 52, four D+/R− participants met the endpoint of CMV disease or infection requiring intervention, of whom two had committee-confirmed CMV syndrome; all recovered with CMV therapy. A total of 5 participants had quantifiable CMV DNAemia through Week 52.

**Conclusion:**

Letermovir was generally well tolerated, and the data support its use for the prevention of CMV disease/infection in adult Japanese KTRs.

**Trial registration:**

ClinicalTrials.gov NCT04129398.

## Introduction

In solid organ transplant (SOT) recipients receiving immunosuppressive drugs, cytomegalovirus (CMV) disease is associated with substantial morbidity and mortality [1, 2]. Direct (CMV syndrome, end-organ disease) and indirect (e.g., allograft rejection, opportunistic infections) effects can present considerable challenges to post-transplant clinical management [1–4]. CMV infection is reported in > 60% of kidney transplant recipients (KTRs) over the first 100 days in the absence of prophylaxis, with the highest risk among CMV-seronegative recipients (R−) with a transplanted kidney from a CMV-seropositive donor (D+) [5, 6]. CMV-seropositive KTRs (R+) are at intermediate risk of CMV infection and disease [7].

Approximately, 1800 kidney transplants are conducted in Japan annually, a majority (~ 90%) of which are living donor transplants. Approximately, 13% of living donor transplants in Japan are reported to be CMV D+/R− [8]. Results of the Japan Academic Consortium of Kidney Transplantation observational cohort study showed CMV seropositivity in 84.6% of KTRs and 94.0% of corresponding donors. The high-risk (D+/R−) group had a high incidence of CMV viremia (70.8%), CMV syndrome (26.9%), and tissue-invasive CMV disease (8.5%), indicating a high disease burden of CMV in Japanese KTRs [9].

The Transplantation Society International CMV Consensus Group’s 2018 guidelines on the management of CMV in solid organ transplantation recommend that high-risk KTRs (i.e., D+/R−) receive 6 months of ganciclovir/valganciclovir prophylaxis or preemptive therapy, while intermediate-risk KTRs (i.e., R+) receive 3 months of valganciclovir prophylaxis or preemptive therapy [2]. The 2022 guideline from the Japan Society for Transplantation recommends valganciclovir prophylaxis in both D+/R− KTRs (180–200 days) and R+ KTRs (90–100 days) [10].

Valganciclovir was approved in 2016 for the prevention of CMV disease in SOT recipients in Japan [11]. However, valganciclovir is associated with myelotoxicity, which can be clinically relevant for SOT recipients receiving concomitant immunosuppressive agents and antibacterial prophylaxis agents that are also myelosuppressive [12, 13]. In addition, valganciclovir requires dose adjustments based on kidney function [14], and valganciclovir-resistant CMV strains have been associated with prolonged treatment and prophylaxis at subtherapeutic exposure [15, 16]. As such, there is an unmet need in Japan for an antiviral with greater tolerability and a lack of cross-resistance with existing therapies for CMV prophylaxis.

Letermovir is a CMV DNA terminase complex inhibitor. This mechanism of action differs from CMV DNA polymerase inhibitors (e.g., valganciclovir), and thus is not expected to be associated with cross-resistance to other anti-CMV agents [17]. Letermovir is approved in over 60 countries for prophylaxis of CMV infection and disease in adult allogeneic hematopoietic stem-cell transplant (HSCT) recipients [18–20]. Approval followed demonstration of a favorable safety profile and clinical efficacy in CMV-seropositive allogeneic HSCT recipients in the pivotal Phase 3 registration study (MK8228-001), which was conducted as a global study including Japan [21]. In another Phase 3 study in CMV D+/R− KTRs (MK8228-002), letermovir was non-inferior to valganciclovir in preventing CMV disease and was generally well tolerated, with fewer leukopenia or neutropenia events in the letermovir group compared with the valganciclovir group [22]. Based on those findings, letermovir was approved for prophylaxis in adult D+/R− KTRs in the United States [18].

The present study was designed to evaluate the safety and tolerability of letermovir for prevention of CMV infection and disease in adult Japanese KTRs. In previous clinical studies, the exposure of letermovir in Japanese or Asian subjects was shown to be higher than in White subjects, and body weight was identified as a key contributing factor [23, 24]. Although the increase was not considered clinically relevant, understanding the safety and tolerability in Japanese KTRs is important. The previous global study in KTRs (MK8228-002) saw limited representation of the Asian population, as only 4 Asian participants (1.4%) received letermovir. The current study supplements the global study by providing additional information on the safety and efficacy of letermovir in Asian KTRs. The study included D+/R− KTRs who are at highest risk of CMV disease, as well as a group of R+ (either D+/R+ or D−/R+) participants who are expected to benefit from prophylaxis. The inclusion of serostatuses in addition to D+/R− is unique to this study.

## Materials and methods

### Study design

This Phase 3 trial (Protocol MK8228-042; ClinicalTrials.gov NCT04129398) is a single-arm, multi-site, open-label study of letermovir safety and efficacy in adult Japanese KTRs. Screening of potentially eligible participants began as early as 1 day before transplantation for participants receiving a kidney from a deceased donor and 14 days prior to transplantation for participants receiving a kidney from a living donor. Eligible participants received the recommended daily adult dose of letermovir 480 mg daily orally (PO), adjusted to 240 mg PO daily with concomitant cyclosporin A (CsA), within 7 days post-transplant for 28 weeks (approximately 200 days). Participants received the intravenous formulation of letermovir if needed. The antiviral activity of letermovir is specific to CMV, but concomitant administration of agents to prevent herpes simplex virus (HSV)/varicella zoster virus (VZV) was not specified by the protocol and was left to the investigators’ discretion. Frequent monitoring of blood concentrations of concomitant drugs including CsA, tacrolimus, and everolimus was recommended to be performed during prophylaxis and at discontinuation of letermovir, with doses adjusted accordingly. After completion of the 28-week prophylaxis period, participants were followed for safety and efficacy through Week 52 (follow-up period). Study visits took place at baseline, Week 1, every 2 weeks from Week 2 to Week 12, and monthly thereafter through Week 52. In addition, a CMV infection/early discontinuation visit was conducted when the investigator suspected CMV disease or intended to initiate CMV therapy for CMV infection, and/or at the time of early discontinuation of letermovir for any reason. Participants were asked to complete the remaining visits of the study through Week 52, even after early discontinuation of study drug.

### Participants

Participants were recruited at four centers in Japan. Eligible participants were Japanese male or female KTRs aged ≥ 18 who were either CMV-seronegative and received an allograft kidney from a documented CMV-seropositive donor (D+/R−) or were CMV-seropositive (R +) and were expected to benefit from 200 days of prophylaxis per the investigator (D+ or D− were allowed for donor CMV serostatus).

The study was designed to enroll 20 participants, of whom at least 10 were to be D+/R−. Key exclusion criteria included prior SOT or HSCT, multi-organ transplant recipients, double kidney transplant recipients, history of CMV disease or suspected CMV disease within 6 months, dialysis or plasmapheresis at the time of allocation, post-transplant renal function of creatinine clearance (CrCl) ≤ 10 mL/min, severe hepatic insufficiency, or both moderate hepatic insufficiency and moderate-to-severe renal insufficiency. Full inclusion/exclusion criteria are listed in the Supplementary Materials.

### Objectives and endpoints

The primary study objective was to evaluate the safety and tolerability of letermovir based on adverse events (AEs) and discontinuation of prophylaxis due to AEs. For clinical evaluation of AEs, all AEs were reported through 14 days after the last dose of prophylaxis. Vital signs, physical examination, 12-lead electrocardiograms, and standard laboratory tests were performed.

Secondary efficacy endpoints were evaluated through Weeks 28 and 52 post-transplant, including CMV disease confirmed by an independent adjudication committee, a composite endpoint of committee-confirmed CMV disease or CMV infection requiring initiation of CMV therapy (i.e., treatment with ganciclovir, valganciclovir, and/or foscarnet at the investigators’ discretion with documented positive results for CMV antigenemia or quantifiable CMV DNAemia per local laboratory assessment), and quantifiable CMV DNAemia (per central laboratory assessment).

CMV disease was assessed at each visit from screening through Week 52. All investigator-reported cases were reported to an independent adjudication committee to be assessed as CMV end-organ disease (organ system involvement with clinical manifestations) or CMV syndrome (infection with prespecified signs/symptoms and/or laboratory criteria in SOT recipients) as defined by the Disease Definitions Working Subgroup of the CMV Drug Development Forum in 2016 [1].

Plasma samples were collected at baseline, Week 2, Week 4, monthly thereafter through Week 52, and at CMV infection and/or early discontinuation visits for CMV DNA PCR (polymerase chain reaction) testing at the central laboratory using the Roche COBAS® AmpliPrep/COBAS® TaqMan® assay (lower limit of quantification, 137 IU/mL). Study investigators were not informed of the CMV DNA PCR test results from the central laboratory but were able to conduct local CMV DNA PCR and/or CMV antigenemia testing at their discretion per institutional standards for clinical management purposes. Letermovir was to be discontinued in the event of suspected or confirmed CMV disease. In the case of asymptomatic CMV viremia, it was the investigators’ decision whether or not to discontinue letermovir to initiate anti-CMV therapy. The study protocol did not define any threshold based on CMV DNA PCR or antigenemia to initiate anti-CMV therapy.

Antiviral resistance to letermovir in prophylaxis failures through Week 52 post-transplant was a prespecified tertiary/exploratory endpoint. Next-generation sequencing (Viroclinics-DDL) of the CMV terminase complex genes UL51, UL56, and UL89 was performed on plasma samples collected from participants at the CMV infection visit, the subsequent visit, and at Week 52 to identify the presence of known letermovir resistance-associated amino acid variants.

The prespecified tertiary/exploratory endpoint of CMV-specific T-cell response was measured using samples collected at screening, Week 12, Week 28, Week 40, Week 52, and at the time of CMV infection and/or early discontinuation visits by a central laboratory using the QuantiFERON®-CMV assay (Qiagen, Hilden, Germany).

### Statistical analysis

There was no formal hypothesis testing for this study.

Safety was analyzed in all participants who received ≥ 1 dose of study drug. AEs were summarized as numbers and percentages of participants who experienced respective events. The development of any of the following four events during prophylaxis was also summarized to evaluate the overall incidence of leukopenia/neutropenia events: an AE of leukopenia, and AE of neutropenia, a central laboratory result for white blood cell count < 3,500 cells/μL, or an absolute neutrophil count < 1,000 cells/μL.

Efficacy was analyzed in the full analysis set (FAS), which included all participants who received ≥ 1 dose of study drug, were D+/R−, D+/R+, or D−/R+, and had no detectable CMV DNAemia (measured by central laboratory) on Day 1. Proportions of participants and associated 95% confidence intervals (CI) were calculated for each of the binary efficacy endpoints by timepoint based on the exact binomial method proposed by Clopper and Pearson [25]. The observed failure (OF) approach was used to handle missing values. With the OF approach, participants who discontinued prematurely from the study for any reason were not considered failures.

The planned sample size (*N* = 20) was derived based on feasibility of enrollment. If a specific AE was not observed in any of the 20 participants, then the true incidence of that event was ≤ 11% with 90% confidence.

## Results

### Participant disposition and baseline characteristics

**Fig. 1:**
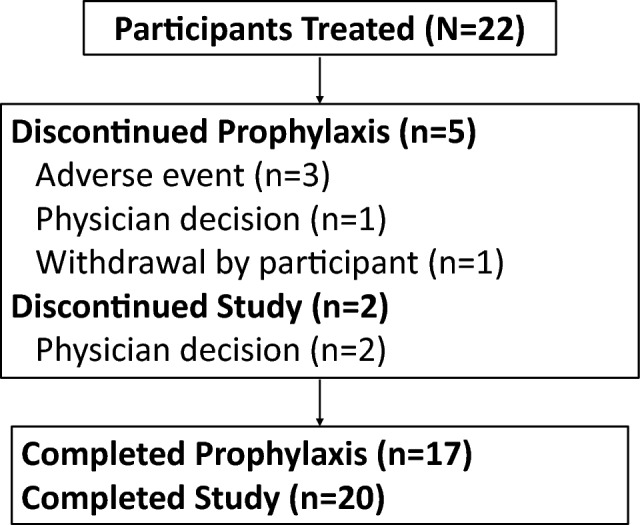
Participant disposition

This study took place from December 27, 2019, to October 6, 2022, at four centers in Japan. Twenty-two participants were enrolled in the study, all of whom received letermovir prophylaxis (Fig. 1). Five participants discontinued letermovir (3 due to an AE, 1 due to physician decision, and 1 due to participant withdrawal) and 2 participants discontinued the study due to physician decision.

Participant baseline characteristics are summarized in Table 1. There were 16 (72.7%) male participants, and the median (range) age was 47.0 years (25 to 70 years). The most common reasons for kidney transplant were diabetic nephropathy and IgA nephropathy. All participants received grafts from living donors. There were 12 (54.5%) CMV D+/R− participants, 9 (40.9%) D+/R+ participants, and 1 (4.5%) D−/R+ participant.

**Table 1:** Baseline demographic and clinical characteristics (all participants as treated)

	Letermovir (*N* = 22)
Age (years), n (%)	
Mean (SD)	48.5 (12.3)
Median (range)	47.0 (25 to 70)
18–35	3 (13.6)
36–50	11 (50.0)
51–64	6 (27.3)
65–74	2 (9.1)
≥ 75	0 (0.0)
Sex, n (%)	
Male	16 (72.7)
Female	6 (27.3)
Race, n (%)	
Asian	22 (100.0)
Primary reason for transplant, n (%)	
Diabetic nephropathy	5 (22.7)
IgA nephropathy	4 (18.2)
Congenital cystic kidney disease	3 (13.6)
Nephrosclerosis	3 (13.6)
End-stage renal disease of unknown etiology	2 (9.1)
Focal segmental glomerulosclerosis	2 (9.1)
Glomerulonephritis chronic	1 (4.5)
Glomerulonephritis rapidly progressive	1 (4.5)
Hemolytic–uremic syndrome	1 (4.5)
Donor type, n (%)	
Living related	21 (95.5)
Living not related	1 (4.5)
Donor/recipient CMV serostatus, n (%)	
Positive/negative (D+/R−)	12 (54.5)
Positive/positive (D+/R+)	9 (40.9)
Negative/positive (D−/R+)	1 (4.5)
ABO-incompatible transplant, n (%)	
Yes	7 (31.8)
No	15 (68.2)
Donor-specific HLA antibody status, n (%)	
Positive	2 (9.1)
Negative	20 (90.9)
Induction immunosuppressive therapy use, n (%)	
Basiliximab	22 (100.0)
Rituximab	11 (50.0)
Maintenance immunosuppressive therapy use, n (%)	
Tacrolimus	22 (100.0)
Cyclosporine	0 (0.0)
Mycophenolate mofetil	17 (77.3)
Azathioprine	1 (4.5)
Everolimus	11 (50.0)
(Methyl) prednisolone	22 (100.0)

ABO, ABO blood group system; D+/R−, donor CMV seropositive, recipient seronegative; D+/R+, donor CMV seropositive, recipient seropositive; D−/R+, donor CMV seronegative, recipient seropositive; IgA, immunoglobulin A; HLA, human leukocyte antigen; SD, standard deviation

Median duration (range) of exposure to letermovir was 195 days (3–200 days). No participants received the intravenous formulation of letermovir.

The most frequently reported drug for induction therapy was basiliximab (used in all participants). Rituximab was used in 11 participants (50.0%). The most frequent immunosuppressants reported during prophylaxis were tacrolimus and methylprednisolone/prednisolone (22 participants; 100%), mycophenolate mofetil (17 participants; 77.3%), and everolimus (11 participants; 50.0%). No participants received CsA as a concomitant medication.

### Safety

In the safety population, which included 22 participants, 90.9% of participants experienced one or more AEs during the prophylaxis period. The most frequent AEs were stomatitis (4 participants; 18.2%), diarrhea, urinary tract infection, decreased neutrophil count, and hyperlipidemia (13.6%; 3 participants each) (Table 2). Four participants (18.2%) had drug-related AEs (leukopenia, diarrhea, nausea, and increased blood alkaline phosphatase). Three participants (13.6%) discontinued letermovir prophylaxis due to AEs (one non-serious AE of leukopenia, considered drug-related, and serious AEs (SAEs) of adenovirus-associated hemorrhagic cystitis and *pneumocystis jirovecii* pneumonia in one participant each, not considered drug-related). A total of 36.4% (8/22 participants) met the prespecified composite safety endpoint of leukopenia/neutropenia events.

**Table 2:** Adverse events through Week 28 post-transplant (safety population)

	Letermovir (*N* = 22)	
Adverse event summary, n (%)		
One or more AE	20 (90.9)	
Drug-related^a^ AE	4 (18.2)	
Serious AE	7 (31.8)	
Serious drug-related AE	0 (0.0)	
Death	0 (0.0)	
Discontinued due to:		
An AE	3 (13.6)	
A drug-related AE	1 (4.5)	
A serious AE	2 (9.1)	
A serious drug-related AE	0 (0.0)	
Adverse events in ≥ 2 participants^b^, n (%)		
Stomatitis	4 (18.2)	
Diarrhea	3 (13.6)	
Urinary tract infection	3 (13.6)	
Decreased neutrophil count	3 (13.6)	
Hyperlipidemia	3 (13.6)	
Leukopenia	2 (9.1)	
Anemia	2 (9.1)	
Nausea	2 (9.1)	
Incisional hernia	2 (9.1)	
Insomnia	2 (9.1)	
Hematuria	2 (9.1)	

^a^Determined by the investigator to be related to the drug

^b^Each participant was counted a single time for each applicable row and column

AE, adverse event

Overall, SAEs were reported in 7 participants (31.8%); none of the SAEs were considered by the investigator to be drug-related. All SAEs resolved (one case of urinary tract infection resolved with sequelae). No deaths were reported.

### Efficacy

The FAS population included 21 participants (one participant who had positive CMV DNA PCR test on Day 1 of the study was excluded). The summary of efficacy through Week 28 and Week 52 post-transplant is shown in Table 3.

**Table 3:** Efficacy outcomes through Week 28 and Week 52 post-transplant (OF approach, FAS population)

	Letermovir total (*N* = 21)		Letermovir D+/R− (*N* = 12)		Letermovir R+ (*N* = 9)	
	n (%)	95% CI^a^	n (%)	95% CI^a^	n (%)	95% CI^a^
CMV disease^b,c^ or started anti-CMV treatment for CMV infection^d^						
Through Week 28 post-transplant	0 (0.0)	(0.0, 16.1)	0 (0.0)	(0.0, 26.5)	0 (0.0)	(0.0, 33.6)
CMV disease^b,c^	0 (0.0)	(0.0, 16.1)	0 (0.0)	(0.0, 26.5)	0 (0.0)	(0.0, 33.6)
Started anti-CMV treatment for CMV infection^d^	0 (0.0)	(0.0, 16.1)	0 (0.0)	(0.0, 26.5)	0 (0.0)	(0.0, 33.6)
Through Week 52 post-transplant	4 (19.0)	(5.4, 41.9)	4 (33.3)	(9.9, 65.1)	0 (0.0)	(0.0, 33.6)
CMV disease^b,c^	2 (9.5)	(1.2, 30.4)	2 (16.7)	(2.1, 48.4)	0 (0.0)	(0.0, 33.6)
Started anti-CMV treatment for CMV infection^d^	4 (19.0)	(5.4, 41.9)	4 (33.3)	(9.9, 65.1)	0 (0.0)	(0.0, 33.6)
Quantifiable CMV DNAemia^e^						
Through Week 28 post-transplant	1 (4.8)	(0.1, 23.8)	1 (8.3)	(0.2, 38.5)	0 (0.0)	(0.0, 33.6)
Through Week 52 post-transplant	5 (23.8)	(8.2, 47.2)	5 (41.7)	(15.2, 72.3)	0 (0.0)	(0.0, 33.6)

Approach to handling missing values: OF approach. With OF approach, participants who discontinued prematurely from the study for any reason were not considered failures

Letermovir total includes all serostatuses (D+/R−, D+/R+, and D−/R+); Letermovir R+ includes D+/R+ and D−/R+

CI, confidence interval; CMV, cytomegalovirus; D+/R+, donor CMV seropositive, recipient seropositive; D+/R−, donor CMV seropositive, recipient seronegative; D−/R+, donor CMV seronegative, recipient seropositive; FAS, full analysis set; OF, observed failure; PCR, polymerase chain reaction

^a^Exact binomial method proposed by Clopper–Pearson method

^b^CMV disease cases confirmed by an independent adjudication committee

^c^CMV disease cases were all CMV syndrome (i.e., CMV infection with ≥ 2 prespecified clinical signs/symptoms and/or laboratory criteria); no cases of CMV end-organ disease (i.e., involvement of ≥ 1 organ system with clinical manifestations) were reported

^d^Anti-CMV treatment (ganciclovir, valganciclovir, or foscarnet) initiated based on at least one positive cell on CMV antigenemia and/or numeric value (not including the result of “Detected but not quantifiable”) of CMV DNA PCR assay performed locally

^e^Quantifiable CMV DNAemia is defined as any detected CMV with a numeric value (≥ 137 IU/mL), based on results from central laboratory

No participants experienced committee-confirmed CMV disease through Week 28 post-transplant. Committee-confirmed CMV disease through Week 52 post-transplant was reported for 2 (9.5%) participants; both cases were reported in D+/R− participants and were assessed as CMV syndrome. There were no cases of CMV end-organ disease. All investigator-reported cases of CMV disease were committee-confirmed without discrepancies in diagnosis.

No participants met the endpoint of committee-confirmed CMV disease or infection requiring intervention through Week 28 post-transplant. Four (19.0%) participants met the endpoint of CMV disease or infection requiring intervention through Week 52 post-transplant; of these, two participants had CMV disease as mentioned previously. All four started CMV treatment and recovered.

Quantifiable CMV DNAemia (> 137 IU/mL) was reported for 1 (4.8%) participant through Week 28 post-transplant. This participant had a single positive CMV DNA PCR test result (236 IU/mL reported by the central laboratory) while on letermovir at Week 16. The test result was negative at a subsequent visit at Week 20 without CMV treatment, and the participant completed 28 weeks of letermovir prophylaxis. Quantifiable CMV DNAemia was reported for a total of 5 (23.8%) participants through Week 52 post-transplant. Apart from the participant described previously with a transient CMV DNAemia through Week 28, all participants received CMV therapy and were counted in the endpoint of CMV disease or infection requiring intervention.

All CMV events meeting the endpoint definition were reported in D+/R− participants; no CMV events were observed in R+ participants.

### Resistance (exploratory endpoint)

Plasma samples were collected and CMV terminase complex genes were analyzed from 4 participants who met the endpoint of CMV disease or infection requiring intervention. No resistance-associated substitutions were detected in any participant at a frequency above the validated assay limit of 5%.

### CMV-specific T-cell response (exploratory endpoint)

The 11 D+/R− participants with evaluable CMV-specific T-cell response data all had negative CMV-specific T-cell response at baseline, and the response at Week 28 post-transplant remained negative for 11/11 (100%) participants. At Week 52 post-transplant, five (45.5%) had positive responses, three of which had met the endpoint of CMV disease or infection requiring intervention.

Among the eight R+ participants with evaluable CMV-specific T-cell response data, two had negative, five had positive, and one had an indeterminate response at baseline. The two participants with negative response at baseline had negative responses at Weeks 28 and 52 post-transplant.

## Discussion

The results obtained from this single-arm, multi-site, open-label study indicate that administration of letermovir 480 mg daily for CMV prophylaxis is generally well tolerated and effective in adult Japanese KTRs who are D+/R−, D+/R+, or D−/R+ for CMV.

In addition to the CMV disease and CMV DNAemia endpoints, which are the standard endpoints of CMV prophylaxis in SOT recipients recommended by the FDA Guidance for Industry [26], the endpoint of CMV disease or infection requiring intervention was included to supplement the CMV disease endpoint by conservatively capturing any intervention for CMV infection which may have progressed to CMV disease if the participant was not given CMV therapy. Overall, CMV disease/infection was well managed while participants were on prophylaxis. Only one case of transient CMV DNAemia was reported during the prophylaxis period, which quickly resolved without intervention. Four cases of CMV infection/disease were reported after cessation of letermovir, but emergence of such late-onset CMV infection/disease is expected from previous CMV prophylaxis trials [22, 27].

This was the first company-sponsored clinical trial to evaluate letermovir prophylaxis in R+ KTRs. While the general recommended duration of CMV prophylaxis in R+ KTRs is 3 months, the Transplantation Society’s international guideline suggests that a longer duration of prophylaxis may be more effective in higher risk patients such as those with ABO-incompatible protocols [2]. The current study enrolled R+ KTRs who were considered to benefit from 200 days of prophylaxis, at the investigators’ discretion. As a result of the study, 10 D+/R+ patients were enrolled, none of whom presented with CMV disease or infection through Week 52 post-transplant. Although the current study lacks a comparator arm, previous studies from Japan reported the incidence of CMV disease to be 0–2% and that of CMV infection to range from 12% to as high as 64% in R+ KTRs without universal prophylaxis. [9, 28, 29] Likewise, studies conducted outside of Japan reported the incidence of CMV disease within 1 year after R+ kidney transplant among those patients receiving valganciclovir 900 mg daily prophylaxis for 100 days to be about 2%. [30, 31] While the lack of CMV events in the current study suggests that R+ KTRs may also benefit from letermovir prophylaxis, which may be effective in suppressing CMV infection, and may be at least as effective as valganciclovir in preventing CMV disease, a firm conclusion or recommendations cannot be made on the optimal duration of prophylaxis for this subgroup of patients.

In the Global Phase 3 Study of letermovir in adult CMV D+/R− KTRs conducted outside of Japan (MK8228-002), letermovir administered through Week 28 post-transplant was non-inferior to valganciclovir for prophylaxis of CMV disease through Week 52 post-transplant, with lower rates of leukopenia or neutropenia events [22]. The feasibility of enrolling a reasonable number of Japanese D+/R− KTRs in this global study was considered low due to the small number of kidney transplantations conducted in Japan. Therefore, the present study was conducted separately using a single-arm design where all subjects received letermovir, so that the amount of data obtained on letermovir in Japanese patients would be maximized. To facilitate data comparisons, the present study was conducted under a similar study protocol as the Global Phase 3 Study during an overlapping time period. Key differences are that the present study is an open-label single-arm study and enrolled R+ participants in addition to D+/R− participants. The inclusion/exclusion criteria and study procedures were almost identical, and the investigator-reported cases of CMV disease were assessed by the same adjudication committee using identical procedures. CMV DNA PCR, resistance, and CMV T-cell response were also tested at the same central laboratories using the same assays.

The safety profile of letermovir was similar in the two studies. Stomatitis was reported at a high frequency in this study while the occurrence was rare in the Global Phase 3 Study [22]. However, KTRs receive concomitant medications that are known to cause stomatitis, most notably everolimus [32, 33], and none of the events were considered drug-related by the investigators.

The incidence of leukopenia/neutropenia was lower in the letermovir group (26.0%) compared to the valganciclovir group (64.0%) in the Global Phase 3 Study [22]. The percentage of leukopenia/neutropenia events reported in this study was slightly higher (36.4%) than that reported for the letermovir group in the Global Phase 3 Study but still lower than the valganciclovir group, suggesting that the decreased risk of myelotoxicity also applies to the Japanese population.

The efficacy in terms of the proportion of participants with committee-confirmed CMV disease through Week 52 post-transplant in the D+/R− population of the present study (committee-confirmed and investigator-reported CMV disease: both 2/12; 16.7%) was similar to that in the Global Phase 3 Study (committee-confirmed CMV disease: 30/289; 10.4%, investigator-reported CMV disease: 50/289, 17.3%) [22]. The proportion of participants with quantifiable CMV DNAemia through Week 52 post-transplant in the D+/R− population of the present study (5/12; 41.7%) was slightly higher than in the Global Phase 3 Study (92/289; 31.8%) [22]. This may be due to the small sample size of the current study, where the contribution of one participant is large. The Global Phase 3 Study did not have the endpoint of CMV disease or infection requiring intervention, so no comparisons were made for this endpoint.

While this study had a small sample size, the results of the exploratory endpoint of viral resistance are consistent with previous letermovir Phase 3 studies [21, 22, 34]. In the Global Phase 3 Study in KTRs, no participants in the letermovir group had letermovir resistance-associated substitutions [22]. Similarly, in this study, no letermovir resistance-associated substitutions were detected. Together, these results suggest that development of resistance to letermovir is not likely to emerge when used as recommended for primary prophylaxis.

CMV-specific T-cell response during letermovir prophylaxis was also evaluated as an exploratory endpoint of the current study. Given the study’s small sample size, it is difficult to interpret the clinical utility of this test method, and results from other clinical trials are pending.

Another difference between the present study and the Global Phase 3 Study is the use of concomitant immunosuppressants. In both studies, tacrolimus and mycophenolate mofetil were two of the most frequently reported concomitant therapies, but the use of concomitant everolimus in the current study was 50%, while its use was minimal in the Global Phase 3 Study [Merck & Co., Inc., Rahway, NJ, USA; Data on File]. As letermovir is a moderate cytochrome P450 3A inhibitor, potential drug–drug interactions (DDI) with letermovir and concomitant immunosuppressants (such as CsA [35], tacrolimus [35], and everolimus [36]) require careful attention in KTRs. In a Phase 1 DDI study where letermovir was coadministered with CsA or tacrolimus, concomitant letermovir increased CsA and tacrolimus exposure by 1.66-fold and 2.42-fold, respectively [35]. In addition, a DDI simulation using physiological-based pharmacokinetic modeling demonstrated that letermovir would likely increase everolimus area under the curve by 2.5-fold [36]. Monitoring of blood concentrations of these concomitant drugs was performed by the investigators during prophylaxis and at discontinuation of letermovir, and the doses of immunosuppressants were adjusted accordingly per the protocol.

There have been reports that the use of everolimus in transplant patients has protective effects against CMV [37, 38]. In the current study, two out of the four events of CMV disease or infection requiring intervention were seen in participants treated with concomitant everolimus. In this trial, no conclusions regarding the potential impact of everolimus on CMV disease can be made in the context of the sizes of the overall trial population and the subset of everolimus recipients.

A limitation of this study is the non-randomized, open-label, single-arm design. In addition, due to the small number of participants, individual cases could have had substantial impact on the overall results. Participant number and design were considered for feasibility of conducting the study in Japan; however, this limits the interpretations that can be made on the safety and efficacy of letermovir.

## Conclusions

The results of this study suggest that administration of letermovir in adult Japanese R+ and D+/R − KTRs for up to 200 days (~ 28 weeks) is generally well tolerated and effective in preventing CMV disease and infection. Letermovir is indicated for prophylaxis for up to 200 days in adult HSCT recipients in Japan, and the data from the current trial support extending the use of letermovir for CMV prophylaxis in adult Japanese KTRs.

## Data Availability

The data sharing policy, including restrictions, of Merck Sharp & Dohme LLC, a subsidiary of Merck & Co., Inc., Rahway, NJ, USA is available at http://engagezone.msd.com/ds_documentation.php. Requests for access to the clinical study data can be submitted through the Engage Zone site or via email to dataaccess@merck.com.
